# Supplementary material for: Interaction of mental comorbidity and physical multimorbidity predicts length-of-stay in medical inpatients
Source: PLoS One. 2023 Jun 22;18(6):e0287234. doi: 10.1371/journal.pone.0287234 (PMC10287009; doi:10.1371/journal.pone.0287234)
Supplement: S1 Table — -P: without psychiatric comorbidity. +P: with psychiatric comorbidity. Sorted by LOS mean difference between cases with and without psychiatric comorbidity. Chapters with total number of cases less than 500 grouped into Z Other: ("III", "VI", "VII", "VIII", "XII", "XIII", "XV", "XVII", "XXI"). Test type U: Mann-Whitney-U. f: common language effect size (value of 0.5 means no significant difference, deviation from 0.5 expresses siginifance), p-value significance: *p ≤ 0.05, **p≤ 0.01, ***p≤ 0.001. (DOCX) [file pone.0287234.s001.docx]

**S1 Table. Length-of-stay (LOS, days) mean and median per main diagnosis chapter.** -P: without psychiatric comorbidity. +P: with psychiatric comorbidity. Sorted by LOS mean difference between cases with and without psychiatric comorbidity. Chapters with total number of cases less than 500 grouped into Z Other: ( "III", "VI", "VII", "VIII", "XII", "XIII", "XV", "XVII", "XXI" ). Test type U: Mann-Whitney-U. f: common language effect size (value of 0.5 means no significant difference, deviation from 0.5 expresses siginifance), p-value significance: *p ≤ 0.05, **p≤ 0.01, ***p≤ 0.001

|  |  | Number of cases | | LOS mean | | | LOS median | | | |  | |
| --- | --- | --- | --- | --- | --- | --- | --- | --- | --- | --- | --- | --- |
| ICD10 Chapter | Main Diagnosis | -P | +P | -P | +P | Diff (sort) | -P | +P | Diff | Test statistic | |  |
| XIX | Injury, poisoning and certain other consequences of external causes | 622 | 140 | 10.7 | 23.0 | **12.3** | 6 | 10 | 4 | U = 31927.0***, f = 0.367 | |  |
| II | Neoplasms | 4095 | 399 | 13.7 | 24.6 | **10.9** | 9 | 16 | 7 | U = 572940.0***, f = 0.351 | |  |
| I | Certain infectious and parasitic diseases | 1308 | 193 | 9.3 | 19.9 | **10.7** | 7 | 10 | 3 | U = 85046.5***, f = 0.337 | |  |
| Z | Other | 1058 | 134 | 6.7 | 16.6 | **9.9** | 5 | 10 | 5 | U = 39938.0***, f = 0.282 | |  |
| XI | Diseases of the digestive system | 3192 | 640 | 8.5 | 18.1 | **9.6** | 6 | 10 | 4 | U = 1384085.0***, f = 0.678 | |  |
| IV | Endocrine, nutritional and metabolic diseases | 1256 | 250 | 7.8 | 17.0 | **9.1** | 6 | 7 | 1 | U = 124479.0***, f = 0.396 | |  |
| IX | Diseases of the circulatory system | 10466 | 1943 | 7.1 | 12.4 | **5.3** | 5 | 7 | 2 | U = 8019640.5***, f = 0.394 | |  |
| XIV | Diseases of the genitourinary system | 750 | 152 | 12.1 | 15.0 | **2.9** | 8 | 11 | 3 | U = 64493.5*, f = 0.566 | |  |
| X | Diseases of the respiratory system | 980 | 313 | 8.0 | 10.0 | **2.0** | 6 | 6 | 0 | U = 154831.0, p = 0.8, f = 0.505 | |  |
| XVIII | Symptoms, signs and abnormal clinical and laboratory findings, not elsewhere classified | 498 | 164 | 6.0 | 6.6 | **0.6** | 4 | 4 | 0 | U = 42717.5, p = 0.4, f = 0.523 | |  |
