# Supplementary material for: Interaction of mental comorbidity and physical multimorbidity predicts length-of-stay in medical inpatients
Source: PLoS One. 2023 Jun 22;18(6):e0287234. doi: 10.1371/journal.pone.0287234 (PMC10287009; doi:10.1371/journal.pone.0287234)
Supplement: S2 Table — These are the underlying numbers for Fig 3. N: number of cases; LOS: length-of-stay; CI95: 95% confidence interval. (DOCX) [file pone.0287234.s002.docx]

**S2 Table. Length-of-stay by age group and presence of mental comorbidity.** These are the underlying numbers for Fig 3. N: number of cases; LOS: length-of-stay; CI95: 95% confidence interval.

| **Age group** | **Mental comorbidity** | **N** | **N ratio within age group** | **LOS mean** | **±LOS CI95** |
| --- | --- | --- | --- | --- | --- |
| 10 | Not present | 192 | 86% | 8.84 | 1.24 |
| 10 | Present | 31 | 14% | 9 | 2.74 |
| 20 | Not present | 1034 | 84% | 8.16 | 0.51 |
| 20 | Present | 196 | 16% | 12.44 | 2.65 |
| 30 | Not present | 1274 | 85% | 8.98 | 0.55 |
| 30 | Present | 228 | 15% | 20 | 5.36 |
| 40 | Not present | 1794 | 82% | 10.19 | 0.62 |
| 40 | Present | 394 | 18% | 16.06 | 2.5 |
| 50 | Not present | 3980 | 82% | 9.96 | 0.37 |
| 50 | Present | 900 | 18% | 19.12 | 2.33 |
| 60 | Not present | 5444 | 86% | 9.43 | 0.28 |
| 60 | Present | 888 | 14% | 17.27 | 1.6 |
| 70 | Not present | 5965 | 87% | 7.94 | 0.21 |
| 70 | Present | 873 | 13% | 13.42 | 1.18 |
| 80 | Not present | 4034 | 85% | 7.81 | 0.21 |
| 80 | Present | 712 | 15% | 9.73 | 0.65 |
| 90 | Not present | 507 | 83% | 7.03 | 0.44 |
| 90 | Present | 106 | 17% | 7.58 | 1.55 |
| 100 | Not present | 1 | 100% | 3 |  |
| 100 | Present | 0 | 0% |  |  |
