# Supplementary material for: Interaction of mental comorbidity and physical multimorbidity predicts length-of-stay in medical inpatients
Source: PLoS One. 2023 Jun 22;18(6):e0287234. doi: 10.1371/journal.pone.0287234 (PMC10287009; doi:10.1371/journal.pone.0287234)
Supplement: S3 Table — These are the underlying numbers for Fig 4. N: number of cases; LOS: length-of-stay; CI95: 95% confidence interval. (DOCX) [file pone.0287234.s003.docx]

**S3 Table. Length-of-stay for increasing Elixhauser score with and without mental comorbidity.** These are the underlying numbers for Fig 4. N: number of cases; LOS: length-of-stay; CI95: 95% confidence interval.

| **Elixhauser score** | **Mental comorbidity** | **N** | **N ratio of total** | **LOS mean** | **±LOS CI95** |
| --- | --- | --- | --- | --- | --- |
| 0 | Not present | 3231 | 11% | 6.48 | 0.2 |
| 0 | Present | 335 | 1% | 6.39 | 0.7 |
| 1 | Not present | 4633 | 16% | 9.52 | 0.29 |
| 1 | Present | 597 | 2% | 9.81 | 1.04 |
| 2 | Not present | 4692 | 16% | 8.38 | 0.26 |
| 2 | Present | 748 | 3% | 10.62 | 1.1 |
| 3 | Not present | 4180 | 15% | 8.05 | 0.29 |
| 3 | Present | 744 | 3% | 14.19 | 1.47 |
| 4 | Not present | 3193 | 11% | 8.42 | 0.3 |
| 4 | Present | 677 | 2% | 15.27 | 1.51 |
| 5 | Not present | 2142 | 8% | 9.54 | 0.4 |
| 5 | Present | 517 | 2% | 17.1 | 1.78 |
| 6 | Not present | 1177 | 4% | 11.22 | 0.74 |
| 6 | Present | 334 | 1% | 20.37 | 3.6 |
| 7 | Not present | 576 | 2% | 13.01 | 1.2 |
| 7 | Present | 188 | 1% | 25.95 | 6.35 |
| 8 | Not present | 249 | 1% | 13.45 | 1.4 |
| 8 | Present | 107 | <1% | 31.64 | 9.75 |
| 9 | Not present | 92 | <1% | 24.28 | 5.81 |
| 9 | Present | 36 | <1% | 56.39 | 23.53 |
| 10 | Not present | 38 | <1% | 24.03 | 10.08 |
| 10 | Present | 28 | <1% | 48.54 | 20.48 |
| 11 | Not present | 14 | <1% | 21.71 | 8.97 |
| 11 | Present | 6 | <1% | 69.67 | 45.36 |
| 12 | Not present | 7 | <1% | 51.86 | 40.34 |
| 12 | Present | 6 | <1% | 84.5 | 88.37 |
| 13 | Not present | 1 | <1% | 5 |  |
| 13 | Present | 3 | <1% | 77.33 | 62.9 |
| 14 | Present | 1 | <1% | 171 |  |
| 15 | Present | 1 | <1% | 139 |  |
