# Supplementary material for: Interaction of mental comorbidity and physical multimorbidity predicts length-of-stay in medical inpatients
Source: PLoS One. 2023 Jun 22;18(6):e0287234. doi: 10.1371/journal.pone.0287234 (PMC10287009; doi:10.1371/journal.pone.0287234)
Supplement: S1 File — R code and output as well as comments for model selection process. (PDF) [file pone.0287234.s005.pdf]

Interaction of mental comorbidity and physical multimorbidity predicts length-of-stay in medical inpatients - Model selection

Load packages and data

```
# Load required packages
require(MASS)  # for glm.nb
```

```
## Loading required package: MASS
```

```
require(broomExtra)  # for tidy_parameters
```

```
## Loading required package: broomExtra
```

```
require(tibble) # for add_row
```

```
## Loading required package: tibble
```

```
require(performance) # for check_overdispersion
```

```
## Loading required package: performance
```

```r
# Set seed for consistent results
set.seed(222)

# Function to load data
load_data = function(dataset_name, keep_columns){
  dataset = read.csv2(file=dataset_name, header=TRUE, sep = ';')

  df = dataset[keep_columns]

  # Check that no column has NA (means missing) values (desired result: 0 for each column)
  print('Check for missing values. Column sums of NA values should be 0:')
  print(colSums(is.na(df)))

  # Change datatypes
  df$gender = as.factor(df$gender)
  df$comorb_F = as.factor(df$comorb_F)
  df$main_diag_level1_chapter = as.factor(df$main_diag_level1_chapter)

  # Check dataframe
  print('Summary of dataframe:')
  print(summary(df))

  return(df)
}
df = load_data(dataset_name, keep_columns)
```

```
## [1] "Check for missing values. Column sums of NA values should be 0:"
##                       los                  Diag_age                    gender
##                         0                         0                         0
##                  comorb_F       elix_score_no_psych comorbidities_psych_count
##                         0                         0                         0
##  main_diag_level1_chapter
##                         0
## [1] "Summary of dataframe:"
##       los              Diag_age      gender     comorb_F     elix_score_no_psych
##  Min.   :  2.000   Min.   : 18.00   1:17219   False:24225   Min.   : 0.000
##  1st Qu.:  3.000   1st Qu.: 54.00   2:11334   True : 4328   1st Qu.: 1.000
##  Median :  6.000   Median : 66.00                           Median : 3.000
##  Mean   :  9.766   Mean   : 63.98                           Mean   : 2.773
##  3rd Qu.: 11.000   3rd Qu.: 77.00                           3rd Qu.: 4.000
##  Max.   :463.000   Max.   :108.00                           Max.   :15.000
##
##  comorbidities_psych_count main_diag_level1_chapter
##  Min.   :0.0000            IX     :12409
##  1st Qu.:0.0000            II     : 4494
##  Median :0.0000            XI     : 3832
##  Mean   :0.1956            IV     : 1506
##  3rd Qu.:0.0000            I      : 1501
##  Max.   :7.0000            X      : 1293
##                            (Other): 3518
```

Functions for model evaluation

```
# Function to describe models
describe_model = function(model_to_describe){
  print('Describing model')
  # Create empty overview dataframe
  overview = data.frame('Variable'=character(),
                        'IRR'=character(),
                        'CI95'=character(),
                        'p-value'=character(),
                        'AIC'=double(),
                        'MAE'=double(),
                        'RMSE'=double())

  # One measure per model
  # Akaike Information Criterion (AIC)
  aic = round(AIC(model_to_describe), 0)
  mae = round(performance_mae(model_to_describe), 2)
  rmse = round(performance_rmse(model_to_describe), 2)

  tp = tidy_parameters(model_to_describe)

  (est <- data.frame(cbind(Estimate = coef(model_to_describe), confint(model_to_describ
e))))
  irr_estimates = exp(est)
  irr_estimates = cbind(Variablename = rownames(irr_estimates), irr_estimates)
  rownames(irr_estimates) = 1:nrow(irr_estimates)

  variables = tp$term
  # Remove all variables that are not fixed variables
  variables = variables[variables != '(Intercept)']

  for(var in variables) {
    pval = tp$p.value[tp$term==var]
    if (pval <= 0.001) {
      sig_stars = '<=0.001***'
    } else if (pval <= 0.01){
      sig_stars = '<=0.01**'
    } else if (pval <= 0.05){
      sig_stars = '<=0.05*'
    } else {
      sig_stars = as.character(round(pval, 2))
    }

    irr_value = as.character(round(irr_estimates$Estimate[irr_estimates$Variablename==va
r], 3))
    ci_low = as.character(round(irr_estimates[['X2.5..']][irr_estimates$Variablename==va
r], 3))
    ci_high = as.character(round(irr_estimates[['X97.5..']][irr_estimates$Variablename==va
r], 3))
    ci_formatted = paste(ci_low, '-', ci_high, sep="")



    overview = overview %>% add_row(!!!setNames(list(var, irr_value, ci_formatted, sig_sta
rs, aic, mae, rmse), names(overview)))
  }
```

```
  return(overview)
}
```

Model selection

Exploratory data analysis involved correlation analysis of several independent variables available in the dataset. Even though only marginally correlated with the dependent variable length-of-stay (los), age at diagnosis (Diag_age) and gender were included in all models as standard demographic information. Main diagnosis ICD-10 chapter (main_diag_level1_chapter) was included in favor over hospital department (organisational unit) since the two variables correlated strongly (Cramer’s V = 0.52) (Cohen 2013), their correlation with los was similar, and main diagnosis would provide more detailed information on the patient’s medical situation. The Elixhauser score excluding the mental comorbidity groups (elix_score_no_psych) was chosen to represent somatic multimorbidity. For mental comorbidity, both the boolean for presence of mental comorbidities of the ICD-10 F-chapter (comorb_F) and the number of comorbidites (comorbidities_psych_count) were tested as alternatives.

Negative binomial regression is particularly suited for the characteristic long-tailed distribution of length-of-stay Rosa and Goldani (2014). To confirm that the distribution of our dataset is also suited to be modeled by negative binomial regression, we first fit a poisson model and check for overdispersion.

Model 1

```
model1 <- glm(los ~ gender + Diag_age + comorb_F + elix_score_no_psych + main_diag_level1_
chapter, family = poisson, data = df)

check_overdispersion(model1)
```

```
## # Overdispersion test
##
##        dispersion ratio =     11.074
##   Pearson's Chi-Squared = 315951.590
##                 p-value =    < 0.001
```

```
## Overdispersion detected.
```

```
multi_model_overview = describe_model(model1)
```

```
## [1] "Describing model"
```

```
## Profiled confidence intervals may take longer time to compute.
##   Use `ci_method="wald"` for faster computation of CIs.
```

```
## Waiting for profiling to be done...
```

```
knitr::kable(multi_model_overview, caption = 'Multivariate Model Overview 1')
```

Multivariate Model Overview 1

Variable	IRR	CI95	p.value	AIC	MAE	RMSE
gender2	1.01	1.003-1.018	<=0.01**	308959	6.44	12.54
Diag_age	0.994	0.994-0.994	<=0.001***	308959	6.44	12.54
comorb_FTrue	1.58	1.566-1.594	<=0.001***	308959	6.44	12.54
elix_score_no_psych	1.17	1.168-1.172	<=0.001***	308959	6.44	12.54
main_diag_level1_chapterII	1.445	1.42-1.47	<=0.001***	308959	6.44	12.54
main_diag_level1_chapterIII	0.883	0.846-0.922	<=0.001***	308959	6.44	12.54
main_diag_level1_chapterIV	0.932	0.911-0.953	<=0.001***	308959	6.44	12.54
main_diag_level1_chapterIX	0.641	0.631-0.652	<=0.001***	308959	6.44	12.54
main_diag_level1_chapterVI	0.977	0.915-1.042	0.48	308959	6.44	12.54
main_diag_level1_chapterVII	0.837	0.656-1.049	0.14	308959	6.44	12.54
main_diag_level1_chapterVIII	0.637	0.483-0.821	<=0.001***	308959	6.44	12.54
main_diag_level1_chapterX	0.73	0.712-0.748	<=0.001***	308959	6.44	12.54
main_diag_level1_chapterXI	0.906	0.89-0.923	<=0.001***	308959	6.44	12.54
main_diag_level1_chapterXII	0.78	0.707-0.857	<=0.001***	308959	6.44	12.54
main_diag_level1_chapterXIII	1.037	0.999-1.077	0.06	308959	6.44	12.54
main_diag_level1_chapterXIV	1.067	1.041-1.093	<=0.001***	308959	6.44	12.54
main_diag_level1_chapterXIX	1.057	1.031-1.084	<=0.001***	308959	6.44	12.54
main_diag_level1_chapterXV	0.663	0.57-0.765	<=0.001***	308959	6.44	12.54
main_diag_level1_chapterXVII	0.584	0.532-0.64	<=0.001***	308959	6.44	12.54
main_diag_level1_chapterXVIII	0.564	0.544-0.583	<=0.001***	308959	6.44	12.54
main_diag_level1_chapterXXI	0.375	0.355-0.396	<=0.001***	308959	6.44	12.54

Model 2

Overdispersion was detected, therefore, negative binomial regression will be used for the following models.

For model comparison, the Akaike Information Criterion (AIC) is suited (Cavanaugh et al.). In addition, the root mean squared error (RMSE) and the mean absolute error (MAE) are computed. The RMSE penalizes large errors more strongly, while the MAE can be interpreted directly as the number of days that the model is wrong by on average. When two models are compared, lower AIC, MAE and RMSE mean better model performance.

```
model2 <- glm.nb(los ~ gender + Diag_age + comorb_F + elix_score_no_psych + main_diag_leve
l1_chapter , data = df)

multi_model_overview = describe_model(model2)
```

```
## [1] "Describing model"
```

```
## Profiled confidence intervals may take longer time to compute.
##   Use `ci_method="wald"` for faster computation of CIs.
```

```
## Waiting for profiling to be done...
```

```
knitr::kable(multi_model_overview, caption = 'Multivariate Model Overview 2')
```

Multivariate Model Overview 2

Variable	IRR	CI95	p.value	AIC	MAE	RMSE
gender2	1.014	0.995-1.033	0.15	180096	6.41	12.6
Diag_age	0.996	0.995-0.996	<=0.001***	180096	6.41	12.6
comorb_FTrue	1.494	1.456-1.532	<=0.001***	180096	6.41	12.6
elix_score_no_psych	1.158	1.152-1.164	<=0.001***	180096	6.41	12.6
main_diag_level1_chapterII	1.492	1.426-1.561	<=0.001***	180096	6.41	12.6
main_diag_level1_chapterIII	0.862	0.779-0.956	<=0.01**	180096	6.41	12.6
main_diag_level1_chapterIV	0.889	0.841-0.94	<=0.001***	180096	6.41	12.6
main_diag_level1_chapterIX	0.627	0.601-0.655	<=0.001***	180096	6.41	12.6
main_diag_level1_chapterVI	0.984	0.833-1.169	0.85	180096	6.41	12.6
main_diag_level1_chapterVII	0.847	0.504-1.514	0.55	180096	6.41	12.6
main_diag_level1_chapterVIII	0.686	0.409-1.207	0.17	180096	6.41	12.6
main_diag_level1_chapterX	0.768	0.725-0.815	<=0.001***	180096	6.41	12.6
main_diag_level1_chapterXI	0.897	0.857-0.94	<=0.001***	180096	6.41	12.6
main_diag_level1_chapterXII	0.838	0.672-1.055	0.12	180096	6.41	12.6
main_diag_level1_chapterXIII	0.994	0.908-1.09	0.9	180096	6.41	12.6
main_diag_level1_chapterXIV	1.119	1.05-1.192	<=0.001***	180096	6.41	12.6
main_diag_level1_chapterXIX	1.045	0.977-1.118	0.2	180096	6.41	12.6
main_diag_level1_chapterXV	0.682	0.493-0.965	<=0.05*	180096	6.41	12.6
main_diag_level1_chapterXVII	0.579	0.477-0.707	<=0.001***	180096	6.41	12.6
main_diag_level1_chapterXVIII	0.575	0.534-0.619	<=0.001***	180096	6.41	12.6
main_diag_level1_chapterXXI	0.386	0.35-0.427	<=0.001***	180096	6.41	12.6

While the MAE and the RMSE are similar for both the general linear model and the negative binomial model, the AIC of the latter is far lower and therefore it is more suited for this data.

Univariate models

One model for each variable is computed to see the predictive power of each individual variable by itself. It serves as an addition to the correlation coefficients to explore the influence of the independent variables on the dependent variable length-of-stay.

```
# Simple models for each individual variable
formulas <- list(
  los ~ gender,
  los ~ Diag_age,
  los ~ comorb_F,
  los ~ comorbidities_psych_count,
  los ~ elix_score_no_psych,
  los ~ main_diag_level1_chapter
)

models = lapply(formulas, glm.nb, data = df)

# Create empty overview dataframe
simple_models_overview = data.frame('Variable'=character(),
                        'IRR'=character(),
                        'CI95'=character(),
                        'p-value'=character(),
                        'AIC'=double(),
                        'MAE'=double(),
                        'RMSE'=double())
for (model_built in models){
  model_overview = describe_model(model_built)
  simple_models_overview = rbind(simple_models_overview, model_overview)
}
```

```
## [1] "Describing model"
```

```
## Profiled confidence intervals may take longer time to compute.
##   Use `ci_method="wald"` for faster computation of CIs.
```

```
## Waiting for profiling to be done...
```

```
## [1] "Describing model"
```

```
## Profiled confidence intervals may take longer time to compute.
##   Use `ci_method="wald"` for faster computation of CIs.
## Waiting for profiling to be done...
```

```
## [1] "Describing model"
```

```
## Profiled confidence intervals may take longer time to compute.
##   Use `ci_method="wald"` for faster computation of CIs.
## Waiting for profiling to be done...
```

```
## [1] "Describing model"
```

```
## Profiled confidence intervals may take longer time to compute.
##   Use `ci_method="wald"` for faster computation of CIs.
## Waiting for profiling to be done...
```

```
## [1] "Describing model"
```

```
## Profiled confidence intervals may take longer time to compute.
##   Use `ci_method="wald"` for faster computation of CIs.
## Waiting for profiling to be done...
```

```
## [1] "Describing model"
```

```
## Profiled confidence intervals may take longer time to compute.
##   Use `ci_method="wald"` for faster computation of CIs.
## Waiting for profiling to be done...
```

```
knitr::kable(simple_models_overview, caption = 'Simple Models Overview')
```

Simple Models Overview

Variable	IRR	CI95	p.value	AIC	MAE	RMSE
gender2	1.005	0.985-1.026	0.62	188128	7.09	13.58
Diag_age	0.994	0.994-0.995	<=0.001***	187805	7.10	13.55
comorb_FTrue	1.722	1.676-1.77	<=0.001***	186480	7.03	13.38
comorbidities_psych_count	1.457	1.43-1.485	<=0.001***	186286	7.02	13.38
elix_score_no_psych	1.11	1.105-1.116	<=0.001***	186092	6.93	13.29
main_diag_level1_chapterII	1.377	1.311-1.446	<=0.001***	185731	6.76	13.33
main_diag_level1_chapterIII	0.852	0.763-0.953	<=0.01**	185731	6.76	13.33
main_diag_level1_chapterIV	0.879	0.827-0.934	<=0.001***	185731	6.76	13.33
main_diag_level1_chapterIX	0.748	0.715-0.783	<=0.001***	185731	6.76	13.33
main_diag_level1_chapterVI	1.081	0.902-1.305	0.41	185731	6.76	13.33
main_diag_level1_chapterVII	0.822	0.47-1.558	0.52	185731	6.76	13.33
main_diag_level1_chapterVIII	0.574	0.331-1.062	0.06	185731	6.76	13.33
main_diag_level1_chapterX	0.797	0.748-0.849	<=0.001***	185731	6.76	13.33
main_diag_level1_chapterXI	0.946	0.899-0.995	<=0.05*	185731	6.76	13.33
main_diag_level1_chapterXII	0.825	0.651-1.059	0.12	185731	6.76	13.33
main_diag_level1_chapterXIII	0.886	0.803-0.978	<=0.05*	185731	6.76	13.33

Variable	IRR	CI95	p.value	AIC	MAE	RMSE
main_diag_level1_chapterXIV	1.179	1.1-1.264	<=0.001***	185731	6.76	13.33
main_diag_level1_chapterXIX	1.218	1.132-1.31	<=0.001***	185731	6.76	13.33
main_diag_level1_chapterXV	0.769	0.543-1.121	0.15	185731	6.76	13.33
main_diag_level1_chapterXVII	0.633	0.515-0.785	<=0.001***	185731	6.76	13.33
main_diag_level1_chapterXVIII	0.578	0.534-0.626	<=0.001***	185731	6.76	13.33
main_diag_level1_chapterXXI	0.412	0.37-0.458	<=0.001***	185731	6.76	13.33

Model 3

Since plotting Elixhauser score and length-of-stay for cases with and without mental comorbidity suggests an interaction between those two variables, the next model includes this interaction.

```
model3 <- glm.nb(los ~ gender + Diag_age + comorb_F*elix_score_no_psych + main_diag_level1
_chapter , data = df)

multi_model_overview = describe_model(model3)
```

```
## [1] "Describing model"
```

```
## Profiled confidence intervals may take longer time to compute.
##   Use `ci_method="wald"` for faster computation of CIs.
```

```
## Waiting for profiling to be done...
```

```
knitr::kable(multi_model_overview, caption = 'Multivariate Model Overview 3')
```

Multivariate Model Overview 3

Variable	IRR	CI95	p.value	AIC	MAE	RMSE
gender2	1.019	1-1.038	0.05	179892	6.4	12.51
Diag_age	0.996	0.995-0.997	<=0.001***	179892	6.4	12.51
comorb_FTrue	1.131	1.081-1.183	<=0.001***	179892	6.4	12.51
elix_score_no_psych	1.138	1.131-1.144	<=0.001***	179892	6.4	12.51
main_diag_level1_chapterII	1.486	1.42-1.554	<=0.001***	179892	6.4	12.51
main_diag_level1_chapterIII	0.856	0.773-0.949	<=0.01**	179892	6.4	12.51
main_diag_level1_chapterIV	0.888	0.84-0.939	<=0.001***	179892	6.4	12.51
main_diag_level1_chapterIX	0.633	0.606-0.66	<=0.001***	179892	6.4	12.51
main_diag_level1_chapterVI	1.014	0.859-1.204	0.87	179892	6.4	12.51
main_diag_level1_chapterVII	0.858	0.512-1.53	0.58	179892	6.4	12.51

Variable	IRR	CI95	p.value	AIC	MAE	RMSE
main_diag_level1_chapterVIII	0.68	0.406-1.193	0.15	179892	6.4	12.51
main_diag_level1_chapterX	0.78	0.735-0.826	<=0.001***	179892	6.4	12.51
main_diag_level1_chapterXI	0.905	0.864-0.947	<=0.001***	179892	6.4	12.51
main_diag_level1_chapterXII	0.825	0.662-1.038	0.09	179892	6.4	12.51
main_diag_level1_chapterXIII	0.993	0.907-1.088	0.88	179892	6.4	12.51
main_diag_level1_chapterXIV	1.132	1.063-1.207	<=0.001***	179892	6.4	12.51
main_diag_level1_chapterXIX	1.053	0.985-1.126	0.13	179892	6.4	12.51
main_diag_level1_chapterXV	0.702	0.507-0.991	<=0.05*	179892	6.4	12.51
main_diag_level1_chapterXVII	0.59	0.487-0.72	<=0.001***	179892	6.4	12.51
main_diag_level1_chapterXVIII	0.588	0.547-0.634	<=0.001***	179892	6.4	12.51
main_diag_level1_chapterXXI	0.391	0.354-0.432	<=0.001***	179892	6.4	12.51
comorb_FTrue:elix_score_no_psych	1.085	1.073-1.097	<=0.001***	179892	6.4	12.51

With the interaction of mental comorbidity and Elixhauser score, all performance measures AIC, MAE and RMSE are lower than for model2.

Model 4

The plot for age at diagnosis also may suggest an interaction between age and mental comorbidity presence. Therefore, here a model with this interaction is computed. However, if compared to the model2 without any interactions, the MAE is equal, the RMSE and the AIC only marginally smaller. The interaction between Elixhauser and mental comorbidity resulted in a larger reduction of AIC.

```
model4 <- glm.nb(los ~ gender + Diag_age*comorb_F + elix_score_no_psych + main_diag_level1
_chapter , data = df)

multi_model_overview = describe_model(model4)
```

```
## [1] "Describing model"
```

```
## Profiled confidence intervals may take longer time to compute.
##   Use `ci_method="wald"` for faster computation of CIs.
```

```
## Waiting for profiling to be done...
```

```
knitr::kable(multi_model_overview, caption = 'Multivariate Model Overview 4')
```

Multivariate Model Overview 4

Variable	IRR	CI95	p.value	AIC	MAE	RMSE
gender2	1.013	0.994-1.032	0.19	180026	6.41	12.58

Variable	IRR	CI95	p.value	AIC	MAE	RMSE
Diag_age	0.997	0.996-0.997	<=0.001***	180026	6.41	12.58
comorb_FTrue	2.265	2.05-2.504	<=0.001***	180026	6.41	12.58
elix_score_no_psych	1.157	1.151-1.163	<=0.001***	180026	6.41	12.58
main_diag_level1_chapterII	1.494	1.428-1.563	<=0.001***	180026	6.41	12.58
main_diag_level1_chapterIII	0.863	0.78-0.957	<=0.01**	180026	6.41	12.58
main_diag_level1_chapterIV	0.885	0.837-0.936	<=0.001***	180026	6.41	12.58
main_diag_level1_chapterIX	0.624	0.598-0.651	<=0.001***	180026	6.41	12.58
main_diag_level1_chapterVI	0.98	0.83-1.164	0.82	180026	6.41	12.58
main_diag_level1_chapterVII	0.842	0.502-1.504	0.54	180026	6.41	12.58
main_diag_level1_chapterVIII	0.676	0.403-1.188	0.15	180026	6.41	12.58
main_diag_level1_chapterX	0.768	0.724-0.815	<=0.001***	180026	6.41	12.58
main_diag_level1_chapterXI	0.893	0.852-0.935	<=0.001***	180026	6.41	12.58
main_diag_level1_chapterXII	0.846	0.679-1.064	0.14	180026	6.41	12.58
main_diag_level1_chapterXIII	0.988	0.902-1.083	0.79	180026	6.41	12.58
main_diag_level1_chapterXIV	1.116	1.047-1.19	<=0.001***	180026	6.41	12.58
main_diag_level1_chapterXIX	1.044	0.976-1.117	0.21	180026	6.41	12.58
main_diag_level1_chapterXV	0.663	0.478-0.938	<=0.05*	180026	6.41	12.58
main_diag_level1_chapterXVII	0.577	0.475-0.704	<=0.001***	180026	6.41	12.58
main_diag_level1_chapterXVIII	0.569	0.529-0.613	<=0.001***	180026	6.41	12.58
main_diag_level1_chapterXXI	0.385	0.348-0.426	<=0.001***	180026	6.41	12.58
Diag_age:comorb_FTrue	0.993	0.992-0.995	<=0.001***	180026	6.41	12.58

Model 5

As an alternative to mental comorbidity presence, the number of mental comorbidities is used.

```
model5 <- glm.nb(los ~ gender + Diag_age +
                    comorbidities_psych_count*elix_score_no_psych +
                    main_diag_level1_chapter
                  , data = df)
multi_model_overview = describe_model(model5)
```

```
## [1] "Describing model"
```

```
## Profiled confidence intervals may take longer time to compute.
##   Use `ci_method="wald"` for faster computation of CIs.
```

```
## Waiting for profiling to be done...
```

```
knitr::kable(multi_model_overview, caption = 'Multivariate Model Overview 5')
```

Multivariate Model Overview 5

Variable	IRR	CI95	p.value	AIC	MAE	RMSE
gender2	1.022	1.003-1.041	<=0.05*	179808	6.46	15.35
Diag_age	0.996	0.995-0.997	<=0.001***	179808	6.46	15.35
comorbidities_psych_count	1.136	1.102-1.171	<=0.001***	179808	6.46	15.35
elix_score_no_psych	1.141	1.135-1.147	<=0.001***	179808	6.46	15.35
main_diag_level1_chapterII	1.494	1.428-1.562	<=0.001***	179808	6.46	15.35
main_diag_level1_chapterIII	0.863	0.78-0.957	<=0.01**	179808	6.46	15.35
main_diag_level1_chapterIV	0.886	0.838-0.936	<=0.001***	179808	6.46	15.35
main_diag_level1_chapterIX	0.633	0.606-0.66	<=0.001***	179808	6.46	15.35
main_diag_level1_chapterVI	1.001	0.848-1.188	0.99	179808	6.46	15.35
main_diag_level1_chapterVII	0.842	0.502-1.501	0.54	179808	6.46	15.35
main_diag_level1_chapterVIII	0.676	0.404-1.186	0.15	179808	6.46	15.35
main_diag_level1_chapterX	0.779	0.735-0.826	<=0.001***	179808	6.46	15.35
main_diag_level1_chapterXI	0.905	0.864-0.948	<=0.001***	179808	6.46	15.35
main_diag_level1_chapterXII	0.818	0.657-1.029	0.08	179808	6.46	15.35
main_diag_level1_chapterXIII	0.986	0.901-1.08	0.76	179808	6.46	15.35
main_diag_level1_chapterXIV	1.135	1.065-1.209	<=0.001***	179808	6.46	15.35
main_diag_level1_chapterXIX	1.036	0.969-1.108	0.31	179808	6.46	15.35
main_diag_level1_chapterXV	0.711	0.515-1.004	<=0.05*	179808	6.46	15.35
main_diag_level1_chapterXVII	0.596	0.492-0.726	<=0.001***	179808	6.46	15.35
main_diag_level1_chapterXVIII	0.582	0.541-0.627	<=0.001***	179808	6.46	15.35
main_diag_level1_chapterXXI	0.388	0.351-0.429	<=0.001***	179808	6.46	15.35
comorbidities_psych_count:elix_score_no_psych	1.044	1.037-1.052	<=0.001***	179808	6.46	15.35

Even though the AIC is slightly lower, the MAE and the RMSE are higher than for model3. Especially the RMSE has increased strongly, meaning that there are larger errors in model4 than in model3. Comorbidity presence is also easier to interpret than a numerical variable for the number of mental comorbidities. Therefore, model3 is chosen as the model to be presented in the manuscript. For further differentiation among mental comorbidities' severance, the individual illnesses are more promising to reveal different effects on the length-of-stay than the pure number of ICD-10 codes. In future work, both somatic and mental multimorbidity should be explored on a more differentiated level, the departments may be considered, and other socio-demographic variables could be explored.

References

Cohen, Jacob. 2013. “Statistical Power Analysis for the Behavioral Sciences.” Journal Article. https://doi.org/10.4324/9780203771587 (https://doi.org/10.4324/9780203771587).

Feliciana Silva, Flávia, Gisele Macedo Da Silva Bonfante, Ilka Afonso Reis, Hugo André Da Rocha, Agner Pereira Lana, and Mariangela Leal Cherchiglia. 2020. “Hospitalizations and Length of Stay of Cancer Patients: A Cohort Study in the Brazilian Public Health System.” Journal Article. *PLOS ONE* 15 (5): e0233293. https://doi.org/10.1371/journal.pone.0233293 (https://doi.org/10.1371/journal.pone.0233293).

Rosa, Regis G., and Luciano Z. Goldani. 2014. “Factors Associated with Hospital Length of Stay Among Cancer Patients with Febrile Neutropenia.” Journal Article. *PLoS ONE* 9 (10): e108969. https://doi.org/10.1371/journal.pone.0108969 (https://doi.org/10.1371/journal.pone.0108969).
