# Supplementary material for: Interaction of mental comorbidity and physical multimorbidity predicts length-of-stay in medical inpatients
Source: PLoS One. 2023 Jun 22;18(6):e0287234. doi: 10.1371/journal.pone.0287234 (PMC10287009; doi:10.1371/journal.pone.0287234)
Supplement: S3 File — (DOCX) [file pone.0287234.s007.docx]

**S6 Supporting Information. Derivation of case simulation calculations.**

Formula as in R code

Model3 <- glm.nb(los ~ gender + Diag_age + comorb_F*elix_score_no_psych + main_diag_level1_chapter, data = df)

Corresponding mathematical formula (<https://stats.oarc.ucla.edu/r/dae/negative-binomial-regression/> )

“The form of the model equation for negative binomial regression is the same as that for Poisson regression. The log of the expected outcome is predicted with a linear combination of the predictors:”

Ln(los) = Intercept +

b1 * gender +

b2 * Diag_age +

b3* comorb_F +

b4* elix_score_no_psych +

b5*comorb_F *elix_score_no_psych +

b6* main_diag(=I)

b7*main_diag(=II) …

“We might be interested in looking at incident rate ratios rather than coefficients. To do this, we can exponentiate our model coefficients.”

los = exp(Intercept +

b1 * gender +

b2 * Diag_age +

b3* comorb_F +

b4* elix_score_no_psych +

b5*comorb_F *elix_score_no_psych +

b6* main_diag(=I) +

b7*main_diag(=II) …

)

Exponentiation of the individual terms leads to multiplication instead of addition:

los = exp( Intercept) *

exp(b1 * gender) *

exp(b2 * Diag_age) *

exp(b3* comorb_F) *

exp (b4* elix_score_no_psych)*

exp(b5*comorb_F *elix_score_no_psych*)

exp(b6* main_diag(=I)) *

exp(b7*main_diag(=II) …

To separate the exponentiated exponents (IRR) from the value of the variables:

**Model formula for direct interpretation of the IRR effects**

los = exp( Intercept) *

exp(b1) ^ (gender) *

exp(b2) ^ (Diag_age) *

**exp(b3) ^ (comorb_F) ***

**exp(b4) ^ (elix_score_no_psych) ***

**exp(b5) ^ (comorb_F *elix_score_no_psych) ***

exp(b6) ^ (main_diag(=I)) *

exp(b7) ^ (main_diag(=II) …
