# Supplementary figures and images for: Interaction of mental comorbidity and physical multimorbidity predicts length-of-stay in medical inpatients
Source: PLoS One. 2023 Jun 22;18(6):e0287234. doi: 10.1371/journal.pone.0287234 (PMC10287009; doi:10.1371/journal.pone.0287234)

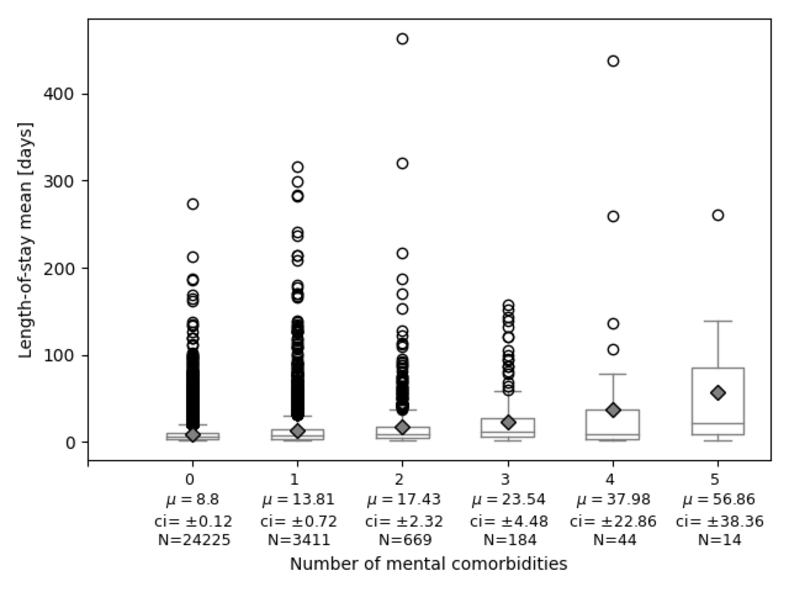

Supplement: S1 Fig — μ: mean length-of-stay, plotted as diamonds; ci: 95% confidence interval, also shown by error bars; N: number of cases. The number of mental comorbidities on the x-axis is displayed up to 5, as the number of cases with 6 or 7 mental comorbidities is less than 10. (TIF) [file pone.0287234.s008.tif]

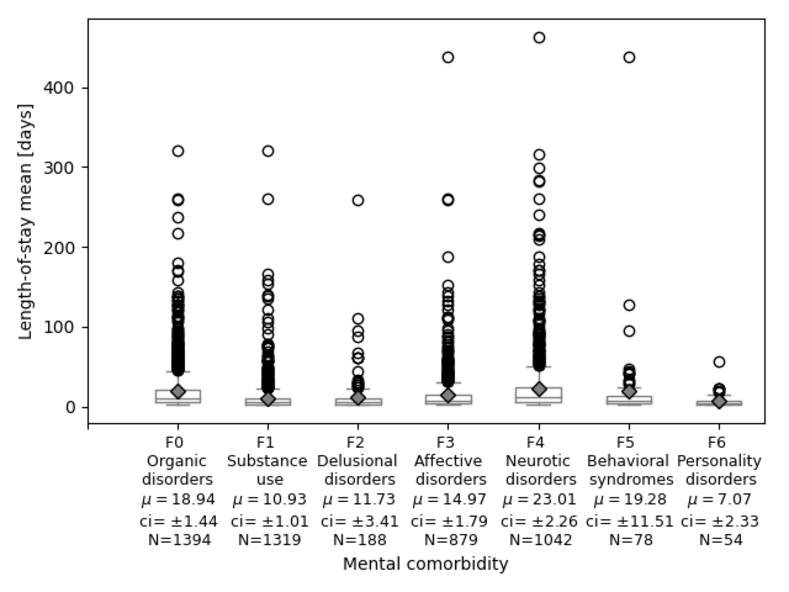

Supplement: S2 Fig — μ: mean length-of-stay, plotted as diamonds; ci: 95% confidence interval, also shown by error bars; N: number of cases that have a diagnosis in the respective F-category (ICD-10 range starting with the characters F0-F6). Cases that have mental comorbidity diagnoses in several F-categories are counted in each one separately and are therefore represented in multiple barsboxes. (TIF) [file pone.0287234.s009.tif]
