# Supplementary material for: Interaction of mental comorbidity and physical multimorbidity predicts length-of-stay in medical inpatients
Source: PLoS One. 2023 Jun 22;18(6):e0287234. doi: 10.1371/journal.pone.0287234 (PMC10287009; doi:10.1371/journal.pone.0287234)
Supplement: Attachment — Submitted filename: PONE-D-23-03643 review.docx [file pone.0287234.s010.docx]

Summary

The authors aimed to investigate the impact of concurrent mental comorbidity and physical multimorbidity on the length of stay in medical inpatients at a maximum-care university hospital. This cross-sectional study has 28,553 inpatients treated in Germany between 2017 and 2019. The authors identified mental health conditions using the ICD-10 chapter and physical comorbidity using the Elixhauser score. Although the research presents an important issue, the authors need to address some issues.

Introduction

The authors wrote, “Moreover, it could be imagined that a future predictive model estimates the expected length-of-stay at the beginning of a hospital stay and supports clinical decisions for early interventions, such as a proactive psychosomatic and psychiatric consultation service.”, I understand this idea is attractive and agree that it is crucial to allocate resources appropriately. However, in practice, principal and additional diagnoses are only recorded after the care is complete, i.e. at the end, so I’m unsure how the model suggested by the authors can be fitted.

Method

The authors should provide more information on the health care system in Germany and, if they need more space, shorten the results section as people from different jurisdictions will need help understanding the system in Germany.

Physical multimorbidity -> need more details on which diagnosis (primary, additional, or all) was used to determine this. I’m guessing it’s all diagnoses, but I need clarification.

Mental comorbidity -> Authors should consider not including F7 in the mental comorbidity as it is an intellectual disability, which is not considered a mental disorder. Generally, people with intellectual disability are known to have a longer stay in the hospital.

Statistical analysis

The authors mentioned that the analysis was performed at the hospitalisation level. Can you please clarify whether you can identify patients from your data? While I agree that the analysis should be performed at the hospitalisation level, I want to know how the authors control for within-patient variation. Records from the same patients could share some characteristics and have a similar outcome; not accounting for it could produce biased results. Also, the authors’ statistical methods assumed data independence to be valid.

Results

Can you please add the 90^th^ percentile for the length of stay in Table 1? The data is skew, so the 90^th^ percentile will help the reader understand the distribution better.

Figures 1 & 2: I believe the box plot with 6 boxes for each mental health group will be more informative than the current graphs.
